# Parental smartphone addiction and adolescent smartphone addiction by negative parenting attitude and adolescent aggression: A cross-sectional study

**DOI:** 10.3389/fpubh.2022.981245

**Published:** 2022-12-01

**Authors:** Eun-Young Doo, Ji-Hye Kim

**Affiliations:** ^1^Nursing Department, Myongji Hospital, Goyang-si, South Korea; ^2^College of Nursing, Woosuk University, Wanju-gun, South Korea

**Keywords:** adolescent, smartphone, parenting attitude, aggression, mediation analysis

## Abstract

**Introduction:**

This study aimed to examine the mediating role of negative parenting attitudes and adolescent aggression in the relationship between parents' and adolescents' smartphone addiction.

**Methods:**

This was a cross-sectional descriptive study that used data from the 2018 Korean Children and Youth Panel Survey. The study involved 2,360 adolescents (1,275 boys, 54.0%, mean age 14.52 ± 0.33 years) and their parents (2,148 mothers, 91.0%), who used smartphones. Adolescents completed questionnaires assessing negative parenting attitudes, aggression, and smartphone addiction while parents completed questionnaires assessing their sociodemographic characteristics and smartphone addiction.

**Results:**

Parents' smartphone addiction was directly and indirectly related to adolescents' smartphone. Additionally, negative parenting attitudes and adolescent aggression played serial mediating roles in the relationship between parents' smartphone addiction and adolescent smartphone addiction.

**Conclusion:**

The findings suggest that it is necessary to consider parental smartphone addiction, parenting attitude, and adolescent aggression, when developing interventions to prevention smartphone addiction among adolescents. Moreover, it highlighted the importance of developing healthy parenting environment that includes parents' healthy smartphone use and positive parenting to prevent adolescents' smartphone addiction.

## Introduction

Smartphones have become an inseparable item for more than 2.7 billion people worldwide for their various features and convenience (1). The users interact with others or their surrounding environments through games, online shopping, education, administrative work, and various social activities on their smartphones, regardless of time and space (2, 3). Smartphones are a convenience in our lives, but they have also brought about a heightened risk of addiction upon excessive use (4). Smartphone addiction has no specific definition in the Diagnostic and Statistical Manual of Mental Disorders-5 (DSM-5), but it is regarded as a behavioral addiction (4, 5). Behavioral addiction refers to the repetition of certain behaviors that cause physiological and psychological difficulties (6) and can be characterized by the obsessive and excessive use of smartphones (7). According to the National Information Society Agency (NIA), 23.3% of smartphone users in Korea showed symptoms of smartphone addiction in 2020, and adolescents accounted for the greatest proportion (39.6%) (8). This figure is 10% points more than other Asian countries such as Japan (22.8–28.0%) and China (22.8 %) (4), which indicates the seriousness of smartphone addiction among Korean adolescents, calling for solutions.

Recent studies have reported that parental factors, one of the environments surrounding adolescents, affect their smartphone use both directly and indirectly, emphasizing the importance of family roles (9–12). Specifically, parents as role models play a critical role in adolescents' problematic behaviors (13, 14). This study aimed to identify the relationship between parents' and adolescents' smartphone addiction. Owing to the lack of previous studies on how smartphone addiction of either parents or adolescents affects each other, the relationship has not been clearly determined; this study assumes that it can be presumed through social learning theory. From the perspective of social learning theory, adolescents' problematic behaviors are learned through interactions with members of society such as their parents, friends, and media (15). Specifically, as families are the major environments where children learn through observation, parents become important role models for them (13). Adolescents who frequently observe risky behaviors of their parents deem them normal, leading to imitation of such behaviors and underestimating the negative consequences (16). Social learning theory has been verified through numerous empirical studies of adolescents' problematic behaviors (14, 15, 17, 18). It has been found that if parents frequently check and compulsively use smartphones at home, their children adopt and imitate their behaviors, which affects the frequency of smartphone use and addictive usage habits (17, 18). In the same context, adolescents whose parents are overly dependent on the Internet are found to be more vulnerable to Internet addiction (19). Therefore, it is hypothesized that parents' smartphone addiction would have a positive correlation with adolescents' smartphone addiction.

Parents' problematic smartphone use may hinder interactions with their children, leading to negative nurturing (9, 14). It has been reported that when parents spend more time on their smartphones, it takes longer for them to react to their children's behaviors or not react at all (20), which has a negative effect on the parent-child relationship (9). In addition, when parents are too preoccupied with smartphones, they become less empathetic and neglectful with their children, and adolescents perceive negative parenting attitudes, such as rejection and coercion (14). According to the Parental Acceptance-Rejection Theory (PAR Theory), formally known as the IPAR Theory, parents' parenting style is related to the psychological adjustment of their adolescent children (21). Specifically, parents' negative parenting attitudes (e.g., cold, unaffectionate, hostile, aggressive, indifferent, neglecting) were found to be highly associated with internalizing, externalizing, and problematic behaviors in their children and adolescents (22). As a result, negative parenting leads to unstable affection between parents and children (23–25), and adolescents show compensatory responses like seeking support in the online space to relieve anxiety and negative emotions caused by deficiency and find psychological stability, ultimately increasing the risk of smartphone addiction (9, 26). Previous studies on the relationship between parenting and adolescent smartphone addiction reported that positive parenting attitudes decreased adolescents' smartphone dependence, and as the attitudes became more negative, the adolescents were more likely to depend on their smartphones (27, 28). Hence, it was hypothesized that parents' negative parenting attitudes would play a mediating role in the relationship between parents' and adolescents' smartphone addiction.

Aggression, a personal factor of adolescents, has been found to be associated with smartphone addiction (29). Aggression refers to behavior in which a person intentionally harms or injures another person (30). Highly aggressive adolescents were observed to have a correlation with violence, drug addiction, and game addiction; as adolescent aggression tends to continue into adulthood, it is critical to make efforts to prevent such a state from developing for both adolescent individuals and society (31, 32). The relationship between parental smartphone addiction and adolescent aggression and smartphone addiction can be explained by the family systems theory and cognitive behavior model. According to the family systems model, stable and healthy family functions are robust implications for adolescents' social, emotional, and behavioral adaptation (33). Family cohesion and conflict are indicators of family functions, and they are associated with adolescents' internalizing or externalizing problems (34, 35). Parents who depend on their smartphones are more likely to be unable to fulfill their parenting responsibilities, be less involved in communication with family members, and lead to problems in family functioning with aggravating conflict (9, 36). Abnormal family functions cause mood problems (angry, depressed, and anxious mood) in adolescents (37), which can influence their aggressiveness (38) and dependency on smartphones (29). Moreover, according to the cognitive-behavioral model developed by Davis (39), an individual's psychopathology such as depression, anxiety, and aggression, influences maladaptive cognitions (e.g., low self-efficacy and negative self-appraisal), becoming the risk factor for pathological Internet usage. Also, previous studies based on the cognitive-behavioral model have found that aggression is an influential factor in Internet and smartphone addiction (29, 40). When parents' problematic smartphone use, such as phubbing, delays reaction to their adolescent children's demands or even leads to no reaction at all, adolescents feel ignored. This in turn decreases their satisfaction in the relationship with their parents, heightening aggressive tendencies (9, 20). In such situations, adolescents tend to rely excessively on their smartphones for emotional desire and support (1, 41). Therefore, we hypothesized that adolescents' aggression plays a mediating role in the relationship between parents' and adolescents' smartphone addiction.

In addition, parents' negative parenting attitudes worsen adolescents' aggression and depression (42, 43). Hence, it was hypothesized that parents' negative parenting attitudes and adolescents' aggression would play a serial mediating role in the relationship between parents' and adolescents' smartphone addiction.

As adolescents have low self-control over the urge to seek pleasure (44) and tend to be more passionate about using mobile devices, they are more vulnerable to smartphone addiction than adults (14). Adolescents' smartphone addiction is associated with not only physical health such as headache, indigestion, sleep disturbance, and blurred vision, but also negative mental health such as depression and anxiety (2, 3, 7, 45). Furthermore, smartphone addiction has led to social issues like declining academic achievement, family conflict, and increased exposure to pornography (46, 47). Therefore, adolescents' surrounding environments need to be considered to understand their behavior. As problematic adolescent behaviors can be attributed to their families, particularly their parents (14), it is important to examine how parental factors are related to adolescents' smartphone addiction. However, as most previous studies on adolescents' smartphone addiction have focused on individual risk factors, there is a lack of research on the structural relationship between parental factors and adolescents' smartphone dependence (48). Therefore, more studies need to be conducted on the mechanisms of multilevel factors that related to adolescents' smartphone addiction. Hence, this study aimed to provide the basis for the prevention of adolescents' smartphone addiction. This was done by examining the mechanism between each factor by focusing on parental factors such as parents' smartphone addiction, negative parenting attitudes, and individual factors (e.g., adolescents' aggression).

The purpose of this study is to identify the mediating role of parental negative parenting attitudes and adolescent aggression in the relationship between parental smartphone addiction and adolescent smartphone addiction and to provide the evidences for the prevention of adolescents' smartphone addiction. The hypotheses of this study are as follows.

Hypothesis 1: Parents' smartphone addiction will be positively related to adolescents' smartphone addiction.
Hypothesis 2: Parents' negative parenting attitudes will play a mediating role in the relationship between parents' smartphone addiction and adolescents' smartphone addiction.
Hypothesis 3: Adolescents' aggression will play a mediating role in the relationship between parents' smartphone addiction and adolescents' smartphone addiction.
Hypothesis 4: Parents' negative parenting attitudes and adolescents' aggression will play a serial mediating role in the relationship between parents' smartphone addiction and adolescents' smartphone addiction.

## Methods

### Design

This study is a cross-sectional, descriptive research study and secondary analysis study utilizing data from the Korean Children and Youth Panel Survey (KCYPS) to verify the mediating effects of negative parenting attitudes and aggression in the relationship between parents' smartphone addiction and adolescents' smartphone addiction.

### Participants

This study used data from the second year of the KCYPS 2018 (49). KCYPS 2018 is a representative panel survey in Korea, where changes in the growth and development of children and adolescents can be examined systematically and multi-dimensionally. Seven surveys were scheduled from 2018 to 2024. The results of the KCYPS 2018 are ideal for understanding the health of adolescents from the perspective of intergenerational transmission, as it studies both generations, parents and children, simultaneously.

The KCYPS 2018 adopted a multi-stage stratified cluster sampling design to construct a systematic and representative sample (49). The second-year survey of the KCYPS 2018 involved students enrolled in the second year of middle school (162 middle schools nationwide) as of 2019. Data were collected between August and November 2019. It was conducted among parent-child pairs. The survey was conducted as a household visit survey using TAPI (tablet assisted personal interview) by trained interviewers. The researchers separated the adolescents and their parents (mother or father) and conducted the survey using two independent questionnaires (student questionnaire and parent questionnaire) at the same time.

Of the total sample of 2,438 adolescents, cases indicating not using a smartphone and cases with missing values for the variables used in the study were excluded; a total of 2,360 adolescents (1,275 males and 1,085 females, mean age 14.52 ± 0.33 years) and parents (2,148 mothers and 212 fathers) were chosen for the survey (Table 1).

**Table 1:** Sociodemographic characteristics (*n* = 2,360).

	**Categories**	***N***	**%**	**M ±SD**
Parental gender	Father	212	9.0	
	Mother	2,148	91.0	
Parental education	≤ High school	821	34.8	
	University	1,409	59.7	
	≥ Graduate school	130	5.5	
Monthly household income	< $1,700	117	5.0	
	$1,700–2,550	163	6.9	
	$2,551–3,400	406	17.2	
	$3,401–4,250	557	23.6	
	$4,251–5,100	450	19.1	
	>$5,100	667	28.3	
Adolescents' gender	Boy	1,275	54.0	
	Girl	1,085	46.0	
Adolescents' age				14.52 ± 0.33

The sample size was calculated using G^*^Power 3.1.9.7. The minimum sample size was 153 when calculated with the median effect size of 0.15, significance level of 0.05, statistical power of 95%, and the number of predictors proposed by Cohen (50).

This study was conducted in accordance with the guidelines of the Declaration of Helsinki and approved by the Institutional Ethics Committee (approval number MJH-2022-05-045) of the hospital where the study was conducted.

### Measurements

#### Sociodemographic characteristics

The gender and educational status of the parents, average monthly household income, and gender of the adolescents were set as the covariates. The educational status of the parents was reclassified into three groups: “high school graduates or below” = 1, “college graduates” = 2, and “graduate school or above” = 3. Average monthly household income was reclassified into groups ranging from “1 = below < 1,700 dollars” to “6 = 5,100 dollars or above.”

#### Smartphone addiction

To measure the smartphone dependence of both parents and adolescents, the “Smartphone addiction self-diagnosis scale” developed by Kim et al. (51) was used, consisting of 15 items and a 4-point Likert scale (“Not at all” = 1 to “Strongly agree” = 4). Of the items, 14 were asked both to parents and adolescents, and one item was modified to suit the participant, asking a different question (for parents: “Work efficiency decreased due to excessive use of smartphones,” and for adolescents: “School grades fell due to excessive use of smartphones”). Three inverse questions were included, and the aggregate score indicated high dependence on smartphones. Cronbach's α was 0.87 upon the development of the scale (51), and was 0.865 and 0.869 for parents and adolescents in this study, respectively.

#### Negative parenting attitudes

To measure the parents' negative parenting attitudes perceived by the adolescents, the “Parents as Social Context Questionnaire for Korean Adolescents: PSCQ-KA” was used, which was developed by Skinner, Johnson, and Snyder (52) and modified by Kim and Lee (53). Here, 12 items were selected, which were about “rejection,” “coercion,” and “chaos.” Each area consisted of four items and was measured on a 4-point Likert scale ranging from “Not at all” (1 point) to “Strongly agree” (four points). Cronbach's α for this study was 0.883.

#### Aggression

To measure adolescent aggression, six items (e.g., There are times when even small things are frowned upon) from the Emotional or Behavioral Problems Scale developed by Cho and Lim (54) were chosen. Each item was rated on a 4-point Likert scale (not at all = 1, disagree = 2, agree = 3, and strongly agree = 4), and Cronbach's α for this study was 0.853.

### Data analysis

SPSS 22.0 and PROCESS macro version 4.0 were used for analysis in the following order. First, frequency analysis was performed to examine the demographic characteristics of the participants. Pearson's correlation analysis was then conducted to determine the correlation between the major variables. In addition, to determine whether negative parenting attitudes and adolescent aggression showed a serial mediating effect in the relationship between parents' and adolescents' smartphone dependence, the PROCESS macro of Hayes was used (model 6). Parents' gender, educational status, monthly average household income, and adolescents' gender were taken as covariates. The significance of individual indirect paths was then identified using bootstrapping. When identifying the significance of the indirect paths, the samples were extracted 5,000 times by applying a 95% confidence interval. If 0 was not included in the confidence interval, the indirect effect was interpreted as significant at the 95% confidence level.

## Results

### Correlation of main variables

The results of the correlation analysis between the independent and dependent variables are presented in Table 2. Adolescents' smartphone addiction was significantly and positively related to their parents' smartphone addiction (*r* = 0.261, *p* < 0.001), negative parenting attitudes (*r* = 0.373, *p* < 0.001), and adolescent aggression (*r* = 0.450, *p* < 0.001).

**Table 2:** Descriptive statistics and correlations between main variables.

**Variables**	***M***	***SD***	**1**	**2**	**3**	**4**
1. Parental smartphone addiction	1.87	0.44	–			
2. Negative parenting attitude	2.01	0.52	0.286^***^	–		
3. Adolescents aggression	2.13	0.46	0.255^***^	0.497^***^	–	
4. Adolescents' smartphone addiction	3.87	0.76	0.261^***^	0.373^***^	0.450^***^	–

M, mean; SD, standard deviation.

***p < 0.001.

### Analysis of mediating effects

To determine whether negative parenting attitudes and adolescent aggression showed a serial mediating effect in the relationship between parents' and adolescents' smartphone dependence, the PROCESS macro was used. Parents' gender, educational status, monthly average household income, and adolescents' gender were taken as covariates, as they are associated with parents' and adolescents' smartphone addiction.

**Figure 1:**
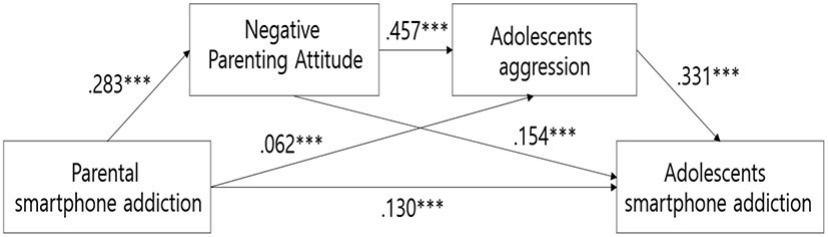
Serial multiple mediation of negative parenting attitude and adolescent aggression in the relationship between parental and adolescents' smartphone addiction with standard path coefficients. ***p < 0.001.

The results of the significance verification of the model paths are presented in Table 3 and Figure 1. Parents' smartphone addiction was positively significant for negative parenting attitudes (*B* = 0.399, *t* = 14.303, *p* < 0.001), adolescent aggression (*B* = 0.062, *t* = 6.222, *p* < 0.001), and adolescents' smartphone addiction (*B* = 0.139, *t* = 6.888, *p* < 0.001). Negative parenting attitudes were also found to be positively significant for adolescent aggression (*B* = 0.206, *t* = 24.802, *p* < 0.001) and adolescents' smartphone addiction (*B* = 0.154, *t* = 8.263, *p* < 0.001), while adolescent aggression was observed to have a positive effect on adolescents' smartphone addiction (*B* = 0.653, *t* = 15.829, *p* < 0.001).

**Table 3:** Results of path analysis.

**Path**	**B**	**se**	***t***	***p***	**LLCI**	**ULCI**
PSA → NPA	0.339	0.024	14.303	< 0.001	0.293	0.386
PSA → AA	0.062	0.010	6.222	< 0.001	0.042	0.082
NPA → AA	0.206	0.008	24.802	< 0.001	0.190	0.222
PSA → ASA	0.139	0.020	6.888	< 0.001	0.099	0.178
NPA → ASA	0.154	0.019	8.263	< 0.001	0.118	0.191
AA → ASA	0.653	0.041	15.829	< 0.001	0.572	0.734

PSA, parental smartphone addiction; NPA, negative parenting attitude; AA, adolescent aggression; ASA, adolescent smartphone addiction.

Bootstrapping was used to verify the significance of indirect effects of negative parenting attitudes and adolescent aggression on the influence of parents' smartphone addiction on adolescents' smartphone addiction. It was conducted by repeatedly extracting the mediator of paths, where parents' smartphone addiction proceeds through negative parenting attitudes and adolescent aggression, and ultimately reaches adolescents' smartphone addiction. The results are presented in Table 4. The path where parents' smartphone addiction passed through negative parenting attitudes and reached adolescent smartphone addiction was found to be significant [*B* = 0.052, CI (0.037–0.069)]. In addition, the path from parents' smartphone addiction to adolescents' smartphone addiction *via* adolescent aggression was significant [*B* = 0.041, CI (0.026–0.055)]. Moreover, the path through which parents' smartphone addiction goes through negative parenting attitudes and adolescent aggression and reaches adolescents' smartphone addiction was found to be significant because it did not include 0 in the 95% confidence interval of the indirect effect [*B* = 0.046, CI (0.036–0.056)].

**Table 4:** Direct and indirect relations in the serial multiple mediation model.

	**B**	**BootSE**	**BootLLCI**	**BootULCI**
Direct effect	0.139	0.020	0.099	0.178
Total indirect effect	0.139	0.011	0.117	0.161
1. PSA → NPA → ASA	0.052	0.008	0.037	0.069
2. PSA → AA → ASA	0.041	0.007	0.026	0.055
3. PSA → NPA → AA → ASA	0.046	0.005	0.036	0.056

PSA, parental smartphone addiction; NPA, negative parenting attitude; AA, adolescent aggression; ASA, adolescent smartphone addiction.

## Discussion

This study attempted to identify whether parents' smartphone addiction is related to adolescents' smartphone addiction through negative parenting attitudes and aggression. The findings showed that negative parenting attitudes and adolescent aggression partially mediated the relationship between parents' and adolescents' smartphone addiction. This implies that parents' smartphone addiction was directly related to adolescents' smartphone addiction while indirectly related to adolescents' smartphone addiction by mediating negative parenting attitudes and adolescent aggression.

Parents' smartphone addiction was directly related to adolescent smartphone addiction, showing a positive correlation, thus supporting Hypothesis 1. This is in line with the findings of previous studies (9, 55) that reported that parents' smartphone dependence directly affects adolescents' dependence on smartphones. Hence, we interpret that the more parents are dependent on smartphones, the more their adolescent children are dependent on smartphones. Moreover, considering other studies (56) that reported that parents' attitudes and usage of smartphones affect their children's smartphone addiction, such results imply that parental education on the correct usage of smartphones needs to take preference, as adolescents tend to imitate their parents' behaviors.

Negative parenting attitudes and adolescent aggression showed a partial mediating effect on the relationship between parents' and adolescents' smartphone addiction. This means that parents' smartphone addiction may not only directly related to adolescents' smartphone addiction, but also may have indirect relationships through the mediating variables, negative parenting attitudes and adolescent aggression; a total of three mediating paths were found to be significant. First, the findings supported Hypothesis 2, as it was found that parents' negative parenting attitudes played a mediating role in the relationship between parents' smartphone addiction and adolescents' smartphone addiction. This can be understood in the same context as the findings (9), which reported that parents' smartphone addiction negatively connected with the parent-child relationship, triggering adolescents' smartphone addiction. When parents are too preoccupied with smartphones, they become less empathetic and more neglectful toward their children. Consequently, children experience negative parenting attitudes, such as rejection, coercion, and inconsistency (14). Thus, adolescents who feel neglected and unsupported by their parents fail to establish a warm and affectionate relationship with them (25); this reflects in the findings that these adolescents tend to rely on their smartphones for emotional support (9, 26). Because the characteristics of parents' smartphone use and parenting attitudes are related factors in adolescents' smartphone use, the findings of this study call for education on parenting attitudes and methods for parents to cope when establishing a strategy for the prevention of smartphone addiction among adolescents.

Second, Hypothesis 3 was supported by the finding that adolescents' aggression mediates parents' smartphone addiction, which was indirectly related to adolescents' smartphone addiction. Such results support the findings of previous studies that parents problematic smartphone use lower the family functions (9, 36) and low family functions increases adolescent aggression (38), consequently increases the risk of adolescent smartphone addiction (29). In short, as parents' overdependence on smartphones results in failure to provide emotional support and basic safety for their children (57), adolescents tend to become cognitively and behaviorally vulnerable in human relationships (58). This eventually hinders building healthy relationships with their family and friends, making them show aggressive behaviors, and ultimately deteriorating their existing relationships with family and friends, which leads to greater dependence on their smartphones (12). Parental smartphone addiction and aggression showed a very weak relationship in this study, however, as aggression is a key variable of adolescents' smartphone dependence, there must be repeated studies on the subject in the future.

Third, Hypothesis 4 was supported as parents' smartphone addiction was indirectly related to adolescents' smartphone addiction by serially mediating negative parenting attitudes and adolescent aggression. This supports the results of previous studies that reported that adolescents with parents who are highly dependent on smartphones experience negative parenting attitudes (14), prolonged negative parenting attitudes build on adolescents' hostile and aggressive behaviors (34, 43), and excessive smartphone use aggravates the relationship with their parents and friends, increasing the time spent on smartphones (34, 35, 39). In addition, adolescent aggression was observed to worsen when their parents used smartphones compulsively, spent less time with them, and were neglectful and hostile; it further worsened when adolescents felt rejected, interfered with, and distrusted by their parents' behaviors (21). Thus, parents' smartphone addiction was found to have both directly and indirectly related to adolescents' smartphone addiction. Therefore, this study calls for the need to devise solutions to reduce parents' smartphone addiction and intervention strategies considering the mediating variables of parenting to prevent smartphone addiction among adolescents.

The significance of the study is as follows. First, this study verified the relationship and paths between each factor in the relationship between parents' and adolescents' smartphone addiction. Specifically, it suggested that three such paths were significant, implying high academic significance. Importantly, the study divided the related factors in adolescents' smartphone addiction into parental and individual factors. Second, since parents' smartphone addiction partly associated to adolescent smartphone addiction, this study suggests that the development and distribution of family education programs are necessary, involving schools and local communities. The content for family education programs should include not only the recommended way of using smartphones and effects of smartphone addiction on individuals and families but also the fact that parents' smartphone addiction and attitudes negatively related to adolescents. It is also necessary to prepare a plan to mediate parental attitudes and adolescent aggression, which have been suggested as leading factors for smartphone addiction among adolescents. Further, it is important to reinforce parental education centering on skills for showing empathy and communication, as well as conflict resolution so that parents can form a good relationship with their children, along with providing information for the improvement of the parent-child relationship and positive parenting. In addition, this study calls for the need to design anger management training or self-control training to reduce aggression in adolescents and monitor the effect on adolescents afterward. These outcomes may be used as primary data for intervention research on the prevention of adolescents' smartphone addiction.

The strength of this study is that it used nationally representative systematic samples recently collected in surveys of parent-child couples, to reduce screening bias and understand adolescent health from the point of view of intergenerational transmission. However, this study has several limitations. First, the present study was a cross-sectional and correlational study. Thus, it was difficult to explain the causal relationship of variables between parents' and adolescents' smartphone addiction. And it was also impossible to control for the baseline levels of mediators and dependent variables. To provide a sounder test of the possible causal validity of the multiple mediation model tested in this study, future studies are required to repeatedly measure mediators and dependent variables and to design a well-controlled longitudinal study. In addition, this study suggests further research to develop intervention programs that include not only adolescents but also parents, and to verify its effects. Second, while this study included some sociodemographic characteristics of parents and adolescents as control variables, a variety of other sociodemographic and environmental factors affect adolescents' smartphone dependence. Hence, further studies should take into account other variables when exploring the association between parents' smartphone addiction and adolescent smartphone addiction. Also, further studies should take a multi-dimensional approach at an individual or group level, including not only parental and individual psychological factors but also factors like school and regional communities, to understand how they affect adolescents' smartphone addiction. Because participants were clustered within middle schools, some shared environmental factors that were not included in the statistical analyses may have artificially increased the strength of the associations among the variables. Thirdly, the questionnaire used in this study was based on a self-reported questionnaire. The participants have individual differences in their responses. Thus, the findings must be interpreted with caution and the use of an objective measurement method such as phone operator data could be considered in future studies. Finally, most of the parents in present study were mothers because of the cultural characteristics of Korean society in which mothers are the main guardians of child rearing. Generally, in Korean society, the father is responsible for the family's financial responsibility and the mother is responsible for raising the children. Since the survey was conducted during the day, the mother in charge of child rearing responded at this time. The gender imbalance of parents may raise concerns about subject bias, but these data are thought to reflect the culture of Korean society well. But, since the influence on adolescents may be different depending on the gender of the parents (59), it is necessary to confirm the difference in influence according to parental gender in future research.

## Conclusion

We found that negative parenting attitudes and adolescent aggression partially mediated the relationship between parents' and adolescents' smartphone addiction. This implies that parents' smartphone addiction was directly related to adolescents' smartphone addiction while indirectly related to adolescents' smartphone addiction by mediating negative parenting attitudes and adolescent aggression. These findings show that parents' smartphone addiction, negative parenting attitudes, and adolescent aggression have both direct and indirect relations on adolescents' smartphone addiction. This study calls for the need to consider parental smartphone addiction, parenting attitude, and adolescent aggression, when developing interventions to prevention smartphone addiction among adolescents. Moreover, it highlighted the importance of developing healthy parenting environment that includes parents' healthy smartphone use and positive parenting to prevent adolescents' smartphone addiction.

## Data availability statement

The raw data supporting the conclusions of this article will be made available by the authors, without undue reservation.

## Ethics statement

The studies involving human participants were reviewed and approved by Myongji Hospital Institutional Review Boards. The participants provided their written informed consent to participate in KCYPS.

## Author contributions

E-YD and J-HK contributed to the conception and design of the study and wrote the first draft of the manuscript. J-HK organized the database and performed the statistical analysis. All authors contributed to manuscript revision, read, and approved the submitted version.

## Conflict of interest

The authors declare that the research was conducted in the absence of any commercial or financial relationships that could be construed as a potential conflict of interest.

## Publisher's note

All claims expressed in this article are solely those of the authors and do not necessarily represent those of their affiliated organizations, or those of the publisher, the editors and the reviewers. Any product that may be evaluated in this article, or claim that may be made by its manufacturer, is not guaranteed or endorsed by the publisher.
